# Grassland coverage change and its humanity effect factors quantitative assessment in Zhejiang province, China, 1980–2018

**DOI:** 10.1038/s41598-022-23210-z

**Published:** 2022-10-31

**Authors:** Linyou Lü, Yan Zhao, Lei Chu, Yongcui Wang, Quanlai Zhou

**Affiliations:** 1 Institute of Sandy Land Amelioration and Utilization, Liaoning Academy of Agricultural Sciences CN, Fuxin, 123000 China; 2 CAS Key Laboratory of Forest Ecology and Management, Institute of Applied Ecology, Chinese Academy of Sciences, 72 Wenhua Road, Shenyang, 110016 People’s Republic of China; 3 Key Laboratory of Pollution Ecology and Environment Engineering, Institute of Applied Ecology, Chinese Academy of Sciences, Shenyang, 110016 China; 4 University of Chinese Academy of Sciences, Beijing, 100039 China

**Keywords:** Grassland ecology, Biodiversity

## Abstract

This study aims to make clear of grassland coverage change and quantitative assessment its effect factors. We collected the data from the National Bureau of Statistics (http://www.stats.gov.cn) and "China 20th Century Land Use/Cover Change (LUCC) Spatio-temporal Platform". Grassland coverage area showed an upward trend from 1980 to 1990, and the grassland coverage area is gradually decreasing from 1990 to 2000, and the grassland coverage area has not changed much from 2000 to 2018. The medium-coverage grassland area has the highest correlation with the total population, and the high-coverage grassland area has the lowest correlation with the total population. Land use types and the composite of gross agricultural output have influence on grassland coverage area. It is hoped that relevant policies should consider land use types and ecological benefits while balancing economic development and urban development.

## Introduction

Vegetation is the main component of terrestrial ecosystems and as an indicator of ecosystem changes. In the world's land area, forest land accounts for about 30%, grassland accounts for 26%, and cultivated land accounts for about 12%^1^. China has the second largest grassland area in the world. The total grassland is 392 million hectares in China, which is about 12% of the world's grassland area and about 41% of China’s national territorial area, which is about twice of China’s arable land^2^. In China, the type of grassland ranks first in the world, mainly including northern grasslands, southern grassy hills and slopes, coastal beaches, wetlands, and natural grasslands in agricultural areas. It includes 18 major categories, 38 subcategories, and more than 1,000 types. The grassland resources also contain extremely rich biodiversity, with more than 7,000 pastures and thousands of animals, making it as the largest biological gene pool in Asia and also the world.

Grassland plays an important role in ecological environment protection and animal husbandry development. Like the grasslands in Europe, China’s land use forms, management objectives, and use systems are becoming increasingly diversified^3^. Grassland has not only made great contributions to preventing soil erosion, purifying chemical fertilizers and pesticides, regulating groundwater, and promoting biodiversity, but also as a basic nutrient for herbivores and ruminants, providing environmental benefits for ensuring the health of grassland animal products. In addition, grassland has aesthetic and entertainment functions, and it can provide functions that other agricultural land use types do not have. In addition, grassland also has an important ecological function of regulating climate^4–6^, for example, grasslands can significantly contribute to climate mitigation while providing substantial additional ecosystem services^7^. Grassland is the only land use type that can accomplish so many tasks and meet so many requirements.

Grasslands are highly vulnerable to climate change or human activities^8^, the research on the relationship between grassland coverage change and its human influencing factors can reflect the scope and degree of influence of natural conditions and human activities on grassland coverage change and has a reference significance for balancing economic development and environmental protection. Grassland is not only an important material basis and means of production for the development of animal husbandry but also an important natural barrier to economic development in southeast china. Zhejiang province is located in the Yangtze River Delta, the transportation is quite convenient, the economic foundation is very well and the economy develops very rapid^9^. Meanwhile, with the rapid development of industrialization and urbanization, the change in land use form has been breathtaking, and human activities have improved the degree of land exploitation and utilization. The natural grassland area in Zhejiang Province is 3 million hm^2^, about 30% of the total land area of the province, of which the available grassland area is 600,000 hm^2^, for about 20% of the total area of natural grassland. Accordingly, there is enormous potential for developing the grassland industry in Zhejiang province^10^.

There are three ways to calculate the grassland coverage, (1) field measurement method, (2) remote sensing estimation method, and (3) integrated measurement method of field measurement and remote sensing estimation^11^. The field measurement method is not suitable for large-scale measurement and measuring alone in various applications, because the measurement range of this method is limited, it is only suitable for the selected field plot. For remote sensing estimation method does not depend on field measurement data, and can reduce the workload and save time, so it is suitable for large-scale grass coverage estimation. At the same time, the field measurement method is an indispensable auxiliary and verification method for modern measurement methods such as remote sensing. Therefore, the comprehensive measurement method of field measurement and remote sensing can obtain more reliable data.

With the rapid development of aerospace science and technology, more and more remote sensing data can be used to monitor land use form^12^. Currently, the most commonly used remote sensing images include Landsat MSS/TM/ETM+, NOAA/AVHRR, and EOS-MODIS. In recent years, satellite SAR, SPOT, CBERS, and other images have also been widely used in research. For global or state-scale land research, NOAA/AVHRR and MODIS data are mainly used. For regional scale, as long as Landsat TM/ETM+ and other high-resolution data are applied.

The change of grassland coverage in Zhejiang Province and its effect factors are of great significance to the development of animal husbandry, the rational development and utilization of land, and the balanced development of the economy and environment. However, there are few studies have been done about this. Therefore, we present the following questions: (1) How did the grassland coverage change in Zhejiang Province from 1980 to 2018? (2) What are the main factors that affect the change in grassland coverage? This study aims to make clear grassland coverage Change and quantitative assessment of its effect factors. Meanwhile, the result of this study will provide a more comprehensive knowledge of the grassland of Zhejiang Province as well as useful suggestions for grassland resource management and sustainable development.

## Materials and methods

### Study area

**Figure 1:**
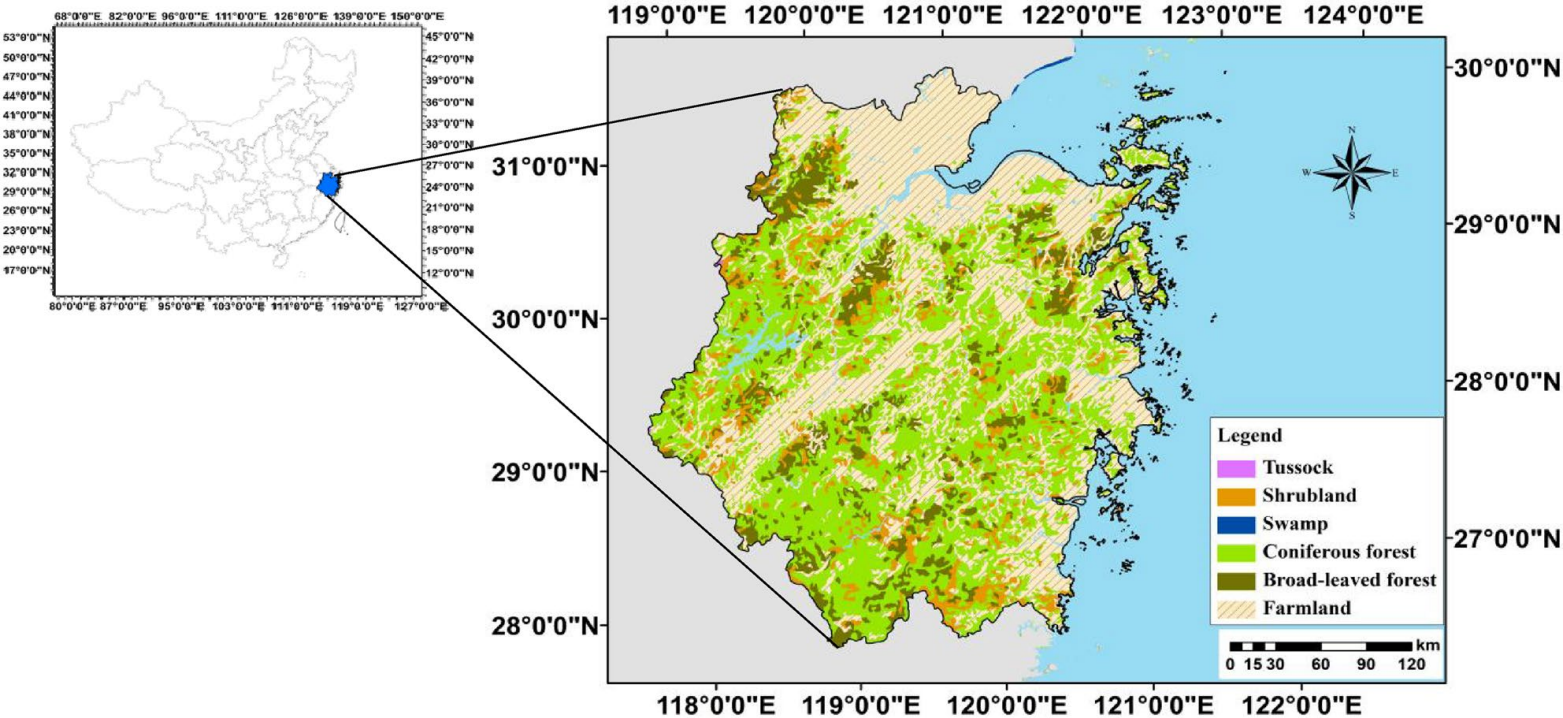
Spatial patterns of digital elevation model over the Zhejiang province, China.

Zhejiang Province (27°02ʹ–31°11ʹN and 118°01ʹ–123°01ʹE) (Fig. 1)^13^, located in southeast China along the East Sea, with an area of around 105 km^2^, has a total land area of 104,141 km^2^ and a population of 58.50 million. The elevation decreases from the southwest to the northeast and varies from 0 to 1800 m, and rivers cross the whole province^14^. Zhejiang experiences a typical northern subtropical monsoon climate, with a mean annual temperature of 15–18 °C and mean annual precipitation of 980–2000 mm^13^. The natural grassland area of Zhejiang Province is 3 million hectares, accounting for about 30% of the land area of the whole province, among which the usable grassland area is 600 thousand hectares, accounting for about 20% of the total natural grassland area of Zhejiang Province.

### Data collection

The total population and composite of gross agricultural output are acquired from the National Bureau of Statistics (http://www.stats.gov.cn), which is an institution directly under the State Council and is in command of national statistics and national economic accounting.

Land use status classification data are acquired from the Geographical Information Monitoring Cloud Platform (http://www.dsac.cn/). These data are based on the domestic land use data produced by Landsat 30 m remote sensing images, according to the method of domestic land use classification, combined with Liu Jiyuan and others in the construction of the "China 20th Century Land Use/Cover Change (LUCC) Spatio-temporal Platform". The LUCC classification system categorizes land use types into six primary categories including cultivated land, woodland, grassland, waters, construction land, unused land, and other 25 secondary categories, including forest land, shrub forest, sparse forest land, other forest land, and high coverage, medium coverage, low coverage grassland. Among them grassland coverage > 50% is defined as high coverage grassland, most of them are natural grassland, improved grassland and mowing grassland, those grassland generally has good water conditions and dense grass cover. Grassland coverage between 20 and 50% is defined as medium coverage grassland, most of them are natural grassland, improved grassland, those grassland generally lacks water and the grass cover is relatively sparse. Low-coverage grassland is the coverage between 5 and 20%, most of which are natural grassland, those grassland generally lacks water, the grass is sparse, and the pastoral utilization conditions are poor.The production of LUCC data is based on the participation of experts, according to the spectral characteristics of the image, combined with the field measurement data, and concerning the relevant geographic maps, the geometric shape, color characteristics, texture characteristics, and spatial distribution of the ground objects were analyzed. The national land use data products are finally obtained through interpretation, and the quality of the data products is checked by combining field surveys and random selection of dynamic patterns for repeated interpretation and analysis. Among them, the classification accuracy of cultivated land data is 85%, and the classification accuracy of other data can reach more than 75%.

### Statistical analyses

Interpret remote sensing image data with ArcGIS software to obtain the data of land use status classification. Use Excel software to organize and summarize the data.

Correlation analyses were performed using Excel, SPSS version 16.0 (SPSS for Windows, Version 16.0, Chicago, Illinois, USA) and CANOCO software (CANOCO 4.5 for Windows)^15^. Except for log transformation the default settings in CANOCO were used for all analyses.

## Results

### Changes of grassland coverage area

**Figure 2:**
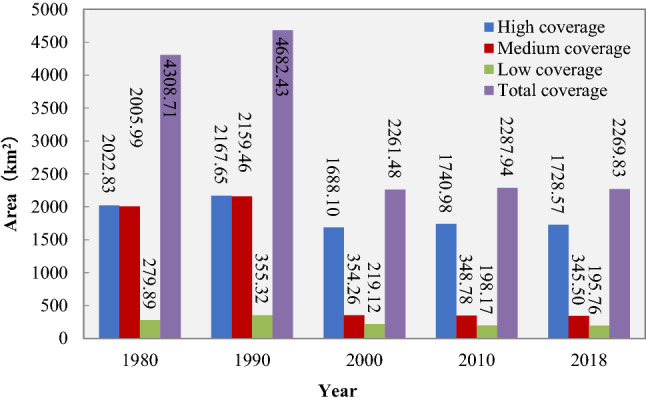
Changes of grassland area with different coverage area.

From 1980 to 2018, the total grassland area, high-coverage grassland area, medium-coverage grassland area, and low-coverage grassland area have basically the same changing trends in Zhejiang Province. The grassland coverage area showed an upward trend from 1980 to 1990, and the grassland coverage area is gradually decreasing from 1990 to 2000, and the grassland coverage area has not changed much from 2000 to 2018 (Fig. 2).

### Correlation between grassland coverage area and total population

**Figure 3:**
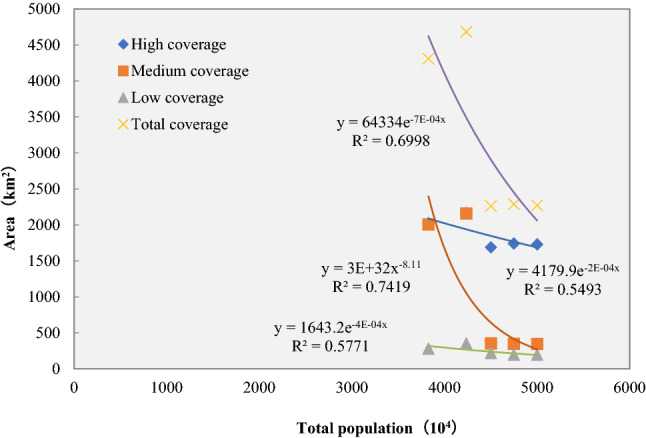
The relationship between grassland coverage area and total population.

The total grassland area has an exponential relationship with the total population (R^2^ = 0.6998), the high-coverage grassland area has an exponential relationship with the total population (R^2^ = 0.5493), the medium-coverage grassland area has a power function relationship with the total population (R^2^ = 0.7419), and the low-coverage grassland area has an exponential relationship with the total population (R^2^ = 0.5771). The medium-coverage grassland area has the highest correlation with the total population, and the high-coverage grassland area has the lowest correlation with the total population (Fig. 3).

### Correlation between grassland coverage area and land use types

**Figure 4:**
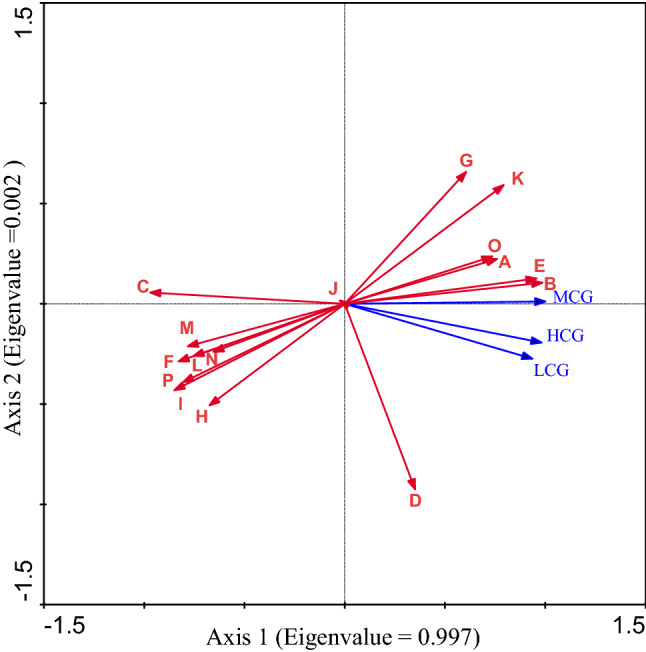
The correlation between the grassland coverage area and land use status classification. HCG = High-coverage grassland area, MCG = Medium-coverage grassland area, LCG = Low-coverage grassland area, A = Paddy field, B = Dry land, C = Forest land, D = Shrub forest land, E = Sparse forest land, F = Other forest land, G = Rivers, H = Lakes, I = Reservoirs and ponds, J = Tidal flats, K = Beach land, L = Towns, M = Rural residential land, N = Industrial and construction land, O = Bare land, P = Bare rock and gravel land.

The redundancy analysis (RDA) results indicated that the correlation between the grassland coverage area and land use status classification had a significant division along the first RDA axis (Eigenvalue = 0.997). High-coverage grassland area, medium-coverage grassland area and low-coverage grassland area had strongly positive correlation with paddy fields (A), dry land (B), shrub forest land (D), sparse forest land (E), beach land (K), bare land (O), and rivers (G), had strongly negative correlation with forest land (C), other forest land (F), lakes (H), reservoirs and ponds (I), towns (L), rural residential land (M), industrial and construction land (N), bare rock and gravel land (P), had week correlated with tidal flats(J) (Fig. 4).

### Correlation between grassland coverage area and the composite of gross agricultural output

**Figure 5:**
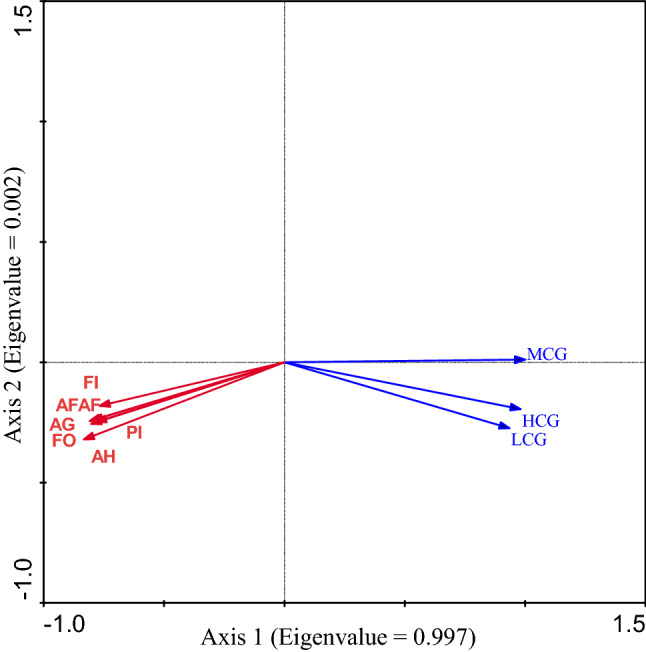
The correlation between the grassland coverage area and composite of gross agricultural output. HCG = High-coverage grassland area, MCG = Medium-coverage grassland area, LCG = Low-coverage grassland area, AFAF = Gross output value of agriculture and forestry, animal husbandry and fishery, AG = Agriculture output, PI = Plantingindustry output, FO = Forestry productionoutput, AH = Animal husbandryoutput, FI = Fisheryoutput.

The RDA results indicated that the correlation between the grassland coverage area and composite of gross agricultural output had a significant division along the first RDA axis (Eigenvalue = 0.997). High-coverage grassland area, medium-coverage grassland area and low-coverage grassland area had a strongly negative correlation with gross output value of agriculture and forestry, animal husbandry and fishery (AFAF), Agriculture output (AG), Planting industry output (PI), Forestry production output (FO), Animal husbandry output (AH), Fishery output (FI) (Fig. 5).

## Discussion

The relationship between land and humans is reflected through the form of land use, the driving factors of land use change mainly include natural factors and human factors^16^. China's reform and opening-up policy began in 1978. With the process of industrialization and urbanization, land use mode gradually changed, and population growth and urban expansion reduced the grassland coverage area^17^. Since 2003, Zhejiang provincial government began to implement the regulations on the protection of prime farmland, and ecological restoration projects in returning farmland to forest, grassland and water body were carried out^18^. Consequently, the downward trend of grassland coverage area was changed. These are the reasons that our study showed that the grassland area in Zhejiang province has shown an upward trend from 1980 to 1990, and the grassland coverage area is gradually decreasing from 1990 to 2000, and the grassland coverage area has not changed much from 2000 to 2018 (Fig. 2). Trend of grassland coverage changes in Zhejiang province was different from Xinjiang province and Sichuan province. Grassland coverage in Xinjiang province has been declining continuously from 2001 to 2015^19^. Some mild degradation and undegraded grasslands were transformed into moderately degraded and severely degraded grasslands in Sichuan province from 2000 to 2010. Therefore, we analyzed the factors that may affect the grassland coverage change, including the total population, the changes in land use status changed and the composite gross agricultural output of Zhejiang. From 1980 to 2018, the total population of Zhejiang Province continued to increase from 38.27 million increased to 50.00 million. This is an important reason that influenced the grassland coverage change. As the population increased, the grassland coverage with decreased trend (Fig. 3), and the total grassland area and medium-coverage grassland area have a high correlation with the total population (R^2^ = 0.70 and 0.74). Some studies have shown that climate change and human activities are two key factors that affect the changes in the grassland ecosystem^20,21^. Among the climatic factors, the change of precipitation is the main reason leading to the change of vegetation coverage^22^, however, the annual precipitation in Zhejiang province 980–2000 mm^14^, so the precipitation is not the restricting factor for grass grows as it does in arid and semi-arid areas.

With the rapid development of urbanization of Zhejiang Province, urban land use, and spatial structures are undergoing significant changes. Based on the analysis of the relationship between the grassland coverage area and land use status classification from 1980 to 2018, showed that land use types have an important effect on grassland coverage area, this found is the same as the study in Canada that land use types and tillage methods are among the major driving factors of soil cover and soil cover change^23^. Our result showed that with the increase of the area of L, M, and N, the area of HCG, MCG and LCG decreased (Fig. 4). With the process of industrialization and urbanization, the demand for L, M and N increases gradually, and grassland is much easier to development and utilization. With population growth and urbanization, promoting the development of agriculture, forestry, animal husbandry and fishery, the gross agricultural output increased. These also explain our result that the area of HCG, MCG and LCG had a strongly negative correlation with the gross output value of agriculture and AFAF, AG, PI, FO, AH, and FI (Fig. 5). It is hoped that relevant policies should consider land use types and ecological benefits while balancing economic development and urban development.

## Conclusion

The results of our study showed that the medium-coverage grassland area has the highest correlation with the total population, and the high-coverage grassland area has the lowest correlation with the total population. Land use types and the composite of gross agricultural output have influence on grassland coverage area. Although there may be some uncertainties about this study's results due to the inaccuracy of remote sensing land use data^24–26^, the research on grassland coverage change and its humanity effect factors quantitative assessment in Zhejiang province have certain reference values for the relevant decision-making of government land use. It is hoped that relevant policies should consider land use types and ecological benefits while balancing economic development and urban development.

## Supplementary Information

Supplementary Information.

## Data Availability

All data generated or analyzed during this study are included in this published article as a Supplementary information file.
