# Supplementary material for: Grassland coverage change and its humanity effect factors quantitative assessment in Zhejiang province, China, 1980–2018
Source: Sci Rep. 2022 Oct 31;12:18288. doi: 10.1038/s41598-022-23210-z (PMC9622824; doi:10.1038/s41598-022-23210-z)
Supplement: Supplementary file 1 — Supplementary Information. [file 41598_2022_23210_MOESM1_ESM.docx]

Supplementary Table 1 Changes of grassland area from 1980 - 2018

|  | Years | Total coverage | High coverage | Medium coverage | Low coverage |
| --- | --- | --- | --- | --- | --- |
| 1 | 1980 | 4308.71 | 2022.83 | 2005.99 | 279.89 |
| 2 | 1990 | 4682.43 | 2167.65 | 2159.46 | 355.32 |
| 3 | 2000 | 2261.48 | 1688.10 | 354.26 | 219.12 |
| 4 | 2010 | 2287.94 | 1740.98 | 348.79 | 198.17 |
| 5 | 2018 | 2269.83 | 1728.57 | 345.50 | 195.76 |

Supplementary Table 2 Output value of agriculture and forestry, animal husbandry and fishery forestry from 1980 - 2018

|  | Years | Gross output value of agriculture and forestry, animal husbandry and fisheryforestry | Agriculture output | Planting industry output | Forestry production | Animal husbandry | Fishery |
| --- | --- | --- | --- | --- | --- | --- | --- |
| 1 | 1980 | 92.67 | 64.23 | 60.52 | 3.61 | 19.39 | 5.44 |
| 2 | 1990 | 331.56 | 199.48 | 163.92 | 16 | 75.18 | 40.9 |
| 3 | 2000 | 1057.07 | 521.31 | 446.15 | 54.48 | 183.94 | 297.36 |
| 4 | 2010 | 2172.86 | 1041.3 | 1041.3 | 119.35 | 448.42 | 522.18 |
| 5 | 2018 | 3157.25 | 1517.96 | 1517.96 | 177.01 | 331.8 | 1043.27 |

Supplementary Table 3 Land use status classification from 1980 - 2018

|  | Years | Paddy field | Dry land | Forest land | Shrub forest land | Sparse forest land | Other forest land | Rivers | Lakes | Reservoirs and ponds | Tidal flats | Beach land | Towns | Rural residential land | Industrial and construction land | Bare land | Bare rock and gravel land |
| --- | --- | --- | --- | --- | --- | --- | --- | --- | --- | --- | --- | --- | --- | --- | --- | --- | --- |
| 1 | 1980 | 25577.26 | 4556.43 | 53429.13 | 1428.76 | 5766.37 | 1610.97 | 1136.41 | 155.10 | 899.69 | 441.12 | 516.88 | 676.55 | 1777.51 | 276.01 | 37.06 | 20.87 |
| 2 | 1990 | 25568.68 | 4536.23 | 53137.20 | 1668.51 | 5551.08 | 1639.52 | 952.46 | 178.43 | 1080.14 | 453.23 | 346.20 | 810.67 | 1785.45 | 321.78 | 28.52 | 25.69 |
| 3 | 2000 | 24872.89 | 3469.11 | 57948.75 | 1431.03 | 4489.80 | 1729.51 | 902.80 | 176.46 | 1212.64 | 432.79 | 271.42 | 1146.01 | 2012.09 | 368.66 | 10.20 | 29.59 |
| 4 | 2010 | 22416.97 | 3335.82 | 56682.07 | 1531.74 | 4805.08 | 1955.90 | 952.62 | 196.16 | 1485.27 | 299.60 | 234.65 | 2535.23 | 2543.37 | 1468.42 | 11.46 | 28.35 |
| 5 | 2018 | 21463.44 | 3165.63 | 56616.44 | 1525.89 | 4758.16 | 1869.41 | 953.97 | 218.02 | 1446.26 | 625.68 | 278.16 | 3107.53 | 2837.21 | 2245.45 | 28.12 | 34.05 |
